# Thermal Stability and Water Content Study of Void-Free Electrospun SPEEK/Cloisite Membrane for Direct Methanol Fuel Cell Application

**DOI:** 10.3390/polym10020194

**Published:** 2018-02-15

**Authors:** Nuha Awang, Juhana Jaafar, Ahmad Fauzi Ismail

**Affiliations:** 1 Advanced Membrane Technology Research Centre (AMTEC), Universiti Teknologi Malaysia, Skudai 81310, Johor Bahru, Malaysia; nuhaawang@yahoo.com (N.A.); 2 Faculty of Chemical and Energy Engineering, Universiti Teknologi Malaysia, Skudai 81310, Johor Bahru, Malaysia

**Keywords:** thermal analysis, wettability, electrospinning, membrane

## Abstract

Void-free electrospun SPEEK/Cloisite15A^®^ densed (SP/e-spunCL) membranes are prepared. Different loadings of Cloisite15A^®^ (0.10, 0.15, 0.20, 0.25 and 0.30 wt %) are incorporated into electrospun fibers. The physico-chemical characteristics (methanol permeability, water uptake and proton conductivity) of the membranes are observed. Thermal stability of all membranes is observed using Thermal Gravimetry Analysis (TGA). The thrree stages of degradation range between 163.1 and 613.1 °C. Differential Scanning Calorimetry (DSC) is used to study the wettability of the membranes. SP/e-spunCL15 shows the lowest freezing bound water of 15.27%, which contributed to the lowest methanol permeability. The non-freezing bound water that proportionally increased with proton conductivity of SP/e-spunCL15 membrane is the highest, 10.60%. It is suggested that the electrospinning as the fabricating method has successfully exfoliated the Cloisite in the membrane surface structure, contributing to the decrease of methanol permeability, while the retained water has led to the enhancement of proton conductivity. This new fabrication method of SP/e-spunCL membrane is said to be a desirable polymer electrolyte membrane for future application in direct methanol fuel cell field.

## 1. Introduction

Direct methanol fuel cell (DMFC) is one of the most outstanding power sources for many ranges of applications, notably portable usages [1]. Owing to the operational stability and high generation of energy, DMFC becomes one of the highlighted power sources nowadays. Polymer electrolyte membrane (PEM) is the heart of DMFC, which is applied to be a medium for methanol to be fed directly, thus PEM optimization has become the focus of a number of research works [2,3].

Nafion^®^ is one of the earliest PEM invented for DMFC [4]. It is chosen due to high proton conductivity [4]. Nevertheless, the disadvantages regarding the conductivity character have limited the application of Nafion^®^ [5]. Since water is the main medium for proton to be delivered, Nafion^®^ has to be properly in hydrated state [6]. If the fully hydrated condition does not occur, the proton conduction in Nafion^®^ becomes slow [7]. High methanol crossover is another significant limitation for Nafion^®^ due to the presence of hydrophilic domains that form from sulfonic groups which allow not only the migration of water and proton but also methanol to permeate [8]. 

To overcome the disadvantages of Nafion^®^, Sulfonated poly (ether ether ketone) (SPEEK) has been chosen due to promising characteristics that have been observed from the previous studies [9,10,11,12]. The research works have proven that SPEEK can be a high potential PEM for DMFC after going through several essential modifications [10]. The modifications that have been done are crosslinking [10], blending with inorganic materials [13] and surface modifications [14].

In the aim of producing a high performance PEM for DMFC, researchers found that a crucial feature that needs to be studied and highlighted is the type of dispersion state. The most favorable one is the exfoliated/delaminated structure [13,14,15]. The introduction of clay as the inorganic additive in SPEEK matrix was one of the earliest trials to produce the exfoliated structure [16]. It was found that the good dispersion of clay brought a positive change in physico-chemical and mechanical properties of PEMs in DMFC application [17].

Nowadays, electrospinning has been applied in wide ranges of applications from medical means to highly structural industries [18]. In DMFC, the application is said to have high ability to produce fine and aligned fibers with good properties that help increase not only physical but also thermal properties [19]. Thus, the new approach in fabricating membrane for DMFC had been applied in this study by introducing electrospun fibers into SPEEK as polymer matrix. The main aim of this work is to study the thermal properties as well as wettability of the membrane in association with the effect of the introduction of electrospun fibers in the polymer matrix. 

## 2. Experimental

### 2.1. Materials

The powder form of Poly (ether ether ketone) (PEEK) was purchased from Vitrex Inc., West Conshohocken, PA, USA. Sulfuric acid of 95–97% was obtained from QRec. The concentrated sulfuric acid was applied as sulfonating agent. The solvent used in the experiment was Dimethylsulfoxide (DMSO) and it was obtained from Sigma-Aldrich (St. Louis, MO, USA). The filler was Cloisite 15A^®^. obtained from Southern Clay Products, Inc. (Gonzales, TX, USA).

### 2.2. Synthesis of Sulfonated Poly (Ether Ether Ketone) (SPEEK)

The sulfonation reaction was handled to provide SPEEK with 64% degree of sulfonation. The experiment was carried out according to the previous study [12]. Fifty grams of PEEK powder were mixed with 1000 mL of PEEK thoroughly for 1 h at room temperature. The temperature was raised to 50 °C so the reaction mixed homogeneously for 3 h. The precipitate of the SPEEK solution was obtained by pouring the solution into ice water bath. The SPEEK was filtered and washed continuously until the pH was 6–7. The SPEEK was then dried in vacuum oven for 24 h at 80 °C.

### 2.3. Preparation of Electrospinning Dope Solution

SPEEK with 10 wt % was firstly dissolved in DMSO. Desired amounts of Cloisite 15A^®^ (0.10, 0.15, 0.20, 0.20 and 0.30 wt %) were dissolved in DMSO at 60 °C under vigorous stirring for 2 h to obtain homogeneous solution, and then added to the SPEEK solution accordingly to produce three samples of dope solution. Each mixture was vigorously stirred for further 24 h at 60 °C to produce a homogeneous solution.

### 2.4. Electrospinning of Nanofibers

During electrospinning, a high voltage power (NF1000 ES93053, MECC CO., LTD. Fukuoka, Japan) was applied to 6 mL of SPEEK/Cloisite solution. The solution was sent to the needle tip via syringe pump to control the solution flow rate. Fibers were obtained on an electrically grounded aluminum foil placed vertically to the needle tip. A voltage of 22.25 kV was applied between the needle and grounded collector at 18.80 cm distance.

### 2.5. Preparation of Void-Free SP/e-spun Cloisite Membrane

**Figure 1:**
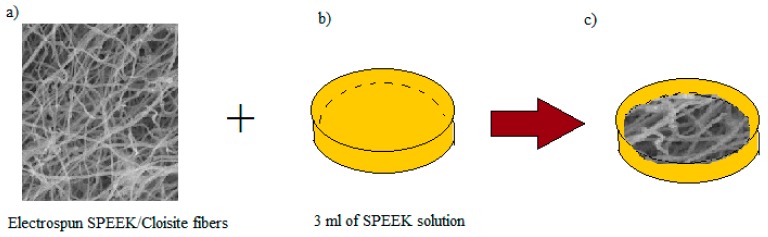
(**a**–**c**) The preparation steps in producing void free dense SP/e-spun CL membranes.

First, 6 mL of SPEEK/Cloisite solution were electrospun according to the parameters decided (see Figure 1a–c). Meanwhile, the SPEEK solution was poured into a petri dish as a base for the membrane, as illustrated in Figure 1b. The solution was then left for 1–5 h for semi solidification process. The electrospun fiber was placed onto the half solidified 3 mL SPEEK solution to cover the surface, as shown in Figure 1c. The electrospun fiber dipped polymer membrane was left for 24 h at room temperature before drying process in the oven for another 24 h at 60 °C. The membrane was casted from petri dish, and then washed by H_2_SO_4_ solution for cross linking process. The membrane was then washed again with water and dried 24 h at 60 °C. The as prepared membranes were designated based on the amount of Cloisite loaded in the SPEEK carrier dope solution. Table 1 summarizes the designation of the electrospun fibers and membrane samples.

### 2.6. Thermogravimetric Analysis (TGA)

Thermal stability test for determining degradation/decomposition temperature of the prepared SP/e-spun CL and parent SPEEK membranes was carried out on a PERKIN ELMER 4000, Perkin Elmer Inc., Petaling Jaya, Selangor, Malaysia. Thermogravimetric Analyzer (TGA). The sample were heated using alumina crucibles under air atmosphere with heating rate of 10 °C/min from room temperature to 500 °C. The instrument was calibrated using melting point of Aluminum, Indium and Lead. The test is applied to determine the weight loss of samples, in particular the elemental decomposition, together with thermal stability which is important to relate with the real DMFC operational temperature that ranges 60–120 °C. From 10 to 15 mg of initial weight of the membrane samples were heated at 210 °C for 30 min to remove moisture and then programmed from 90 to 900 °C at a rate of 10 °C·min^−1^ under nitrogen atmosphere.

### 2.7. Differential Scanning Calorimetry (DSC) Analysis 

Thermal properties test for determining glass transition temperature (*T*_g_) and wettability of the prepared membranes was performed on Mettler-Toledo Analyzer (DSC822C/600, Mettler Toledo, Shah Alam, Malaysia). 

For *T*_g_ determination, sample of 10 to 20 g were used. The analysis was performed and recorded a preliminary cycle of thermal by heating the specimen at the rate of 10 °C·min^−1^ in nitrogen atmosphere from ambient to 160 °C. The analysis was held for 10 min. The sample was quenched cool to 90 °C below the transition peak of interest. The temperature was held until a steady state was reached. The heating was repeated at rate of 10 °C until the entire desired peak was observed.

For wettability test, 5 mg of samples were annealed at 50 °C for 10 min under nitrogen atmosphere then quenched and scanned at heating rate of 10 °C·min^−1^. The free water content of membrane sample was defined under nitrogen atmosphere at 5 °C·min^−1^ between temperatures that ranged −50 °C to 30 °C.

### 2.8. Water Uptake Measurement

Water uptake as one of the key parameters for proton exchange membrane has been measured in this study for different as developed membranes. The water content in the membranes was observed by measuring the difference of membranes’ weight in wet and dry conditions. The adequate hydration of electrolyte membranes is critical to achieve a better fuel cell performance [13]. After drying the membranes in vacuum oven at 60 °C for 48 h, the membranes with approximately 1.5 cm in diameter was soaked in deionized water overnight at room temperature. The surface moisture was then removed using absorbent paper and the weight was recorded for dried sample. The water uptake was calculated as follows:

Water (%) = (G_w_ – G_d_)/G_d_ × 100 (1)

where G_w_ and G_d_ are the weight of the wet and dry membranes, respectively.

### 2.9. Proton Conductivity Measurement

**Figure 2:**
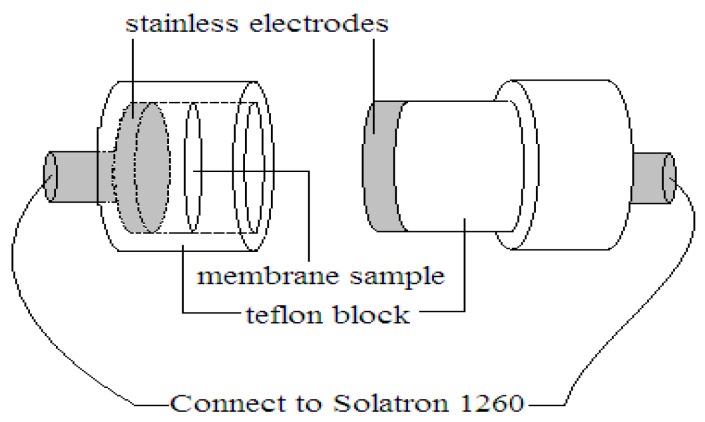
Schematic diagram of the proton conductivity cell.

Transverse proton conductivities of electrospun nanocomposite membranes were generated by impedance spectroscopy over a frequency range of 1 to 10^7^ Hz with 50 to 500 mV oscillating voltage (model Solartron 1260 Gain phase Analyser, AMETEK, Inc., Leicester, UK). The hydrated membrane having 15 mm diameter was sandwiched between two stainless steel electrodes, as shown in Figure 2.

The conductivity ∂ of samples in the transverse direction is measured from the impedance data, using the relationship as follows:

(2) $$\partial =\frac{d}{RS}$$

*∂* = proton conductivity (S·cm^−1^)
*d* = membrane thickness (cm)
*R* = resistance (ohm) (the value was derived from the low intersection of the high frequency semi-circle on a complex impedance plane with the Re (Z) axis)
*S* = membrane cross section area (cm^2^)

Three replicate were collected and averaged. It is important to ensure all tested membranes were soaked in water for 24 h.

### 2.10. Methanol Permeability Measurement

The test was handled by observing the permeability of methanol in electrospun nanocomposite membranes to determine barrier properties of the membranes [17]. This study employed the diaphragm diffusion cell in which methanol (1 Molar)–water mixture and water were contained in two compartments that were separated by a test membrane. The concentration of methanol was chosen as 1 Molar because several studies found methanol permeability of SPEEK based membrane increased with the increasing of methanol concentration. The experiment was handled at room temperature. After 3 h of the methanol permeability test, 1 mL was sampled from both compartments to determine methanol concentration by Perkin Elmer High Performance Liquid Chromatography (HPLC). The methanol permeability value was calculated using the following equation:

(3) $$P=\alpha \times \frac{V_{B}}{A}\times \frac{L}{C_{A}}$$

where *P* is the methanol permeability (cm^2^·s^−1^), and *α* is the slope of methanol permeation of the sample (M/s). The slope is calculated as follows:

*α* = *C*_B_ (*t*)/(*t* − *t*_o_) (4)

where

*C_B_ (t)* = concentration of methanol in compartment B at time, t (M)
*t_o_* = time lag, related to the diffusivity (s)
*V_B_* = volume of water in compartment B (cm^3^) = 200 cm^3^
*A* = membrane cross-section area (cm^2^)
*L* = membrane thickness (cm)
*C_A_* = concentration of methanol in compartment *A* at time, t (M) = 1 M

### 2.11. Tensile Test

The tensile strength of the membrane was measured using mechanical testing system MTS (LRX 5 kN, Lloyd Instruments, West Sussex, UK) according to ASTMD638. The gauge length and width of dumbbell tensile specimens were 25 and 5 mm, respectively. The specimen was placed between the grips of the testing machine, and the speed of testing was set at the rate of 0.5 mm·min^−1^. Five specimens were taken from each type of membrane for the measurement and their average value was calculated.

### 2.12. Scanning Electron Microscopy Analysis (SEM)

The morphology behavior of SPEEK, its nanocomposite membranes and electrospun fibers for high magnification was investigated using scanning electron microscopy (SEM) (JSM-6390LV, JEOL USA, Inc., Peabody, MA, USA) was used. Specimens for the morphological analysis were prepared by freezing the dry membrane samples in liquid nitrogen and breaking them to produce a cross-section. Fresh cross-sectional cryogenic fractures of the membranes were vacuum sputtered with a thin layer of gold before SEM examination.

## 3. Results and Discussion

### 3.1. Thermal Stability Study of Void-Free SP/e-spunCL Membranes

The fuel cells that exhibit a better performance when it is operated at a high temperature is said to possess a very high thermal stability. Hence, thermal characteristic is an important subject to be studied to obtain a high performance fuel cell. Thermogravimetric analysis for the void-free SP/e-spunCL membranes were performed with the aims of finding the thermal stability behavior and to observe the vulnerable functional group that evolve when heat applied.

**Figure 3:**
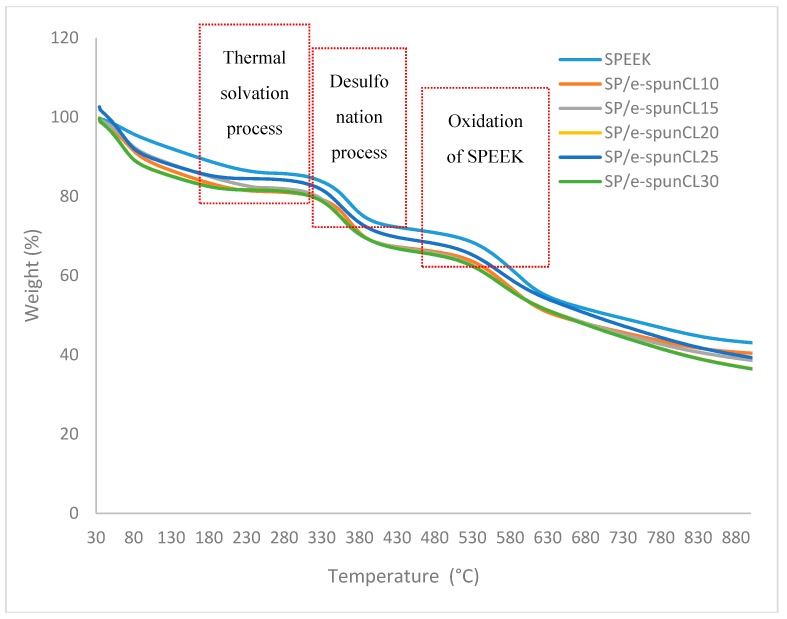
TGA of SPEEK and SP/e-spunCL membranes with various formulations.

Figure 3 shows the degradation stages for all samples. Three steps of degradation occurred as a result of thermal solvation process, desulfonation and oxidation of SPEEK as polymer matrix [20].

Table 2 tabulates the degradation temperature (*T*_d_) and weight loss of SP/e-spunCL membranes. The first weight loss (*T*_d1_) occurred at temperature ranging from 163.1 to 209.1 °C caused by the loss of absorbed water molecules and the range also represents the decomposition of Cloisite 15A ^®^[21]. The weight loss in the T_d1_ ranged from 9.444 to 20.558 wt %. The absorption of water can be explained as a polymer with sulfonic acid group such as SPEEK is naturally hydrophilic and will absorb moisture from surrounding [22]. The absorbed water molecules mostly exist in a bound state rather than being in free molecule state [23].

The second weight loss (*T*_d2_) that occurred at 388.1–410 °C with the mass loss of 12.703–15.306 wt % can be related to the crosslinked bonds breakage which is the –CO–O– bonds that resulted from the desulfonation of sulfonic acid [24]. In addition, sulfonic groups tend to decompose at the earliest at 288 °C due to thermal instability [24].

In the third weight loss region (*T*_d3_) at temperature ranging from 564.1 to 617.1 °C with mass loss from 11.126 to 17.914% occurred due to the breakdown of SPEEK backbone [25]. As shown in the SPEEK degradation curve in Figure 2, SPEEK started to degrade from 163.1 to 564.1 °C, whereas the other membranes only begin to decompose at around 192.1–617.1 °C. This result showed that all membranes have good thermal stability for the DMFC application, which all was recorded to be thermally stable within the range of 60 to 120 °C of the DMFC operating temperature. 

In addition, it was also realized that the degradation temperature increased as the addition of Cloisite increased. Referring to the results, the SP/e-spunCL membranes showed higher degradation temperature in contrast to pure SPEEK membrane. The SP/e-spunCL10 showed degradation temperature of 602.1 °C and, for e-spunCL30, it was 617.1 °C, which is, respectively, 38 and 53 °C increased with 10 and 30 wt % of the Cloisite. The decomposition temperatures for others also increased: SP/e-spunCL10 (606.1 °C), SP/e-spunCL15 (610.1 °C) and SP/e-spunCL25 (613.1 °C).

The above results indicate that the membranes with addition of inorganic fillers/additives, i.e. Cloisite, were thermally more stable than their pure polymer. This is in agreement with previous research that was carried out for polymer/clay nanocomposites [26,27,28]. It can be discussed that the impact of Cloisite had clearly noted as mass transport barrier and superior insulation against the volatile compound resulted during the degradation of polymer when heat was applied [29]. 

In addition, Cloisite is also reported to be an element that causes the formation of layered carbonaceous char during degradation [29]. This can be reaffirmed by the observation of the Cloisite content of SP/e-spunCL membranes remaining as residue after heating [30]. The residuals can be explained by the inorganic materials that are more thermally stable in the ranges of temperature where the organic compounds (SPEEK) were already degraded into volatile compounds [30]. As for the TGA, by means of the degradation and thermal stability of the membranes study, SP/e-spunCL membranes have been thermally improved as compared to pure SPEEK membrane.

### 3.2. Wettability Analysis of the Void-Free SP/e-spunCL Membranes

**Figure 4:**
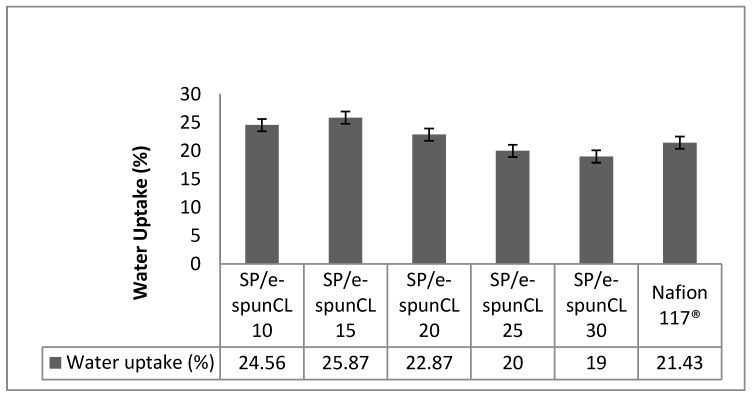
Water uptake of all SP/e-spunCL membranes and Nafion 117^®^.

**Figure 5:**
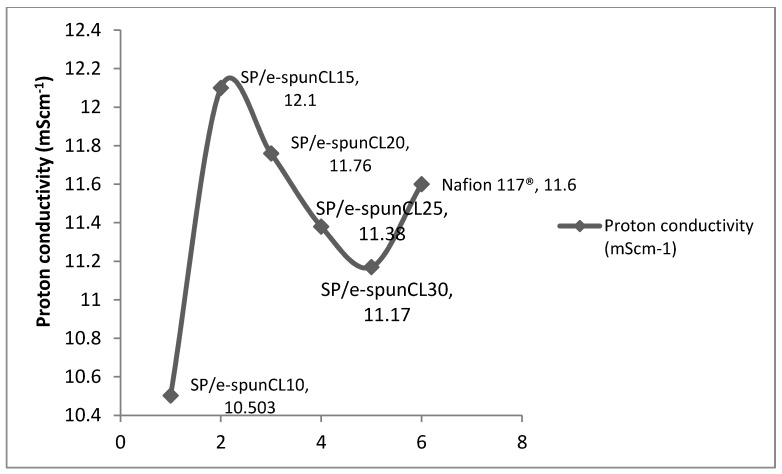
Proton conductivity of Nafion117^®^ and all membrane samples at room temperature.

**Figure 6:**
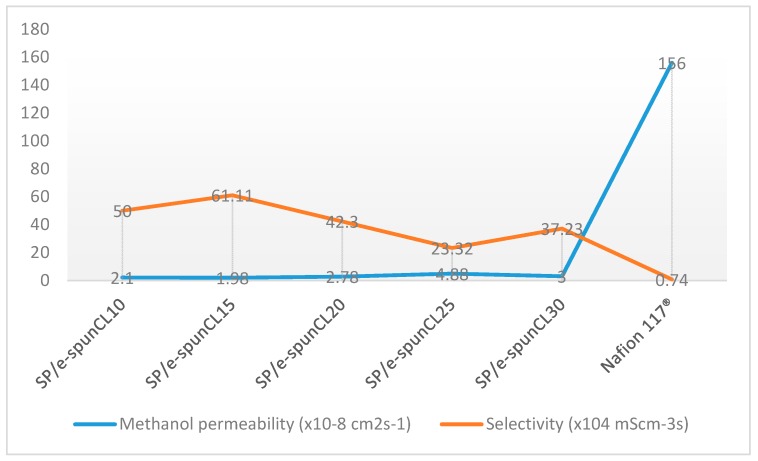
Methanol permeability of Nafion117^®^ and all membrane samples at room temperature.

Water uptake played an important role as it affects membrane transport properties, for example water diffusion coefficient and proton conductivity. Low proton conductivity will increase cell resistance and low water diffusion coefficient will hinder methanol to diffuse to catalyst sites in taking reaction. The drawbacks lead to the fuel low efficiency. Hence, water uptake is an important element in DMFC operation. Figure 4, Figure 5 and Figure 6, shows the physico-chemical properties (water uptake, Proton conductivity and methanol permeability) of SP/e-spunCL membranes and Nafion^®^.

The dependence of water uptake on membrane dehydration conditions may have important implications in the use of these membranes in DMFC. Less water is taken up by the membrane during cell operation, a decrease in the maximum obtainable membrane conductivity occurs, since the conductivity depends roughly linearly on membrane water uptake as proton carrier. Figure 5 shows the proton conductivity of all SP/e-spunCL membranes.

In Figure 5, SP/e-spunCL15 had the highest conductivity values with 12.12 mS·cm^−1^. The lowest conductivity was SP/e-spunCL10, at 10.30 mS·cm^−1^. As the amount of Cloisite added in electrospun SP/Cloisite increased, the proton conductivity increased up to 12.12 mS·cm^−1^. The increasing value of proton conductivity did not happen for the following formulations: SP/e-spunCL25 and SP/e-spunCL30. The different results are not only related to water uptake which played role as proton carrier but also the fineness of fibers which embedded into the membrane. Finer fibers with less beaded structures show higher proton conductivity of the membrane, promoted by the interactions of electrospun fibers with SPEEK polymer matrix [31]. 

S direct methanol fuel cell also requires that the membranes have low methanol permeability. Figure 6 shows methanol permeability for all SP/e-spunCL membranes.

**Figure 7:**
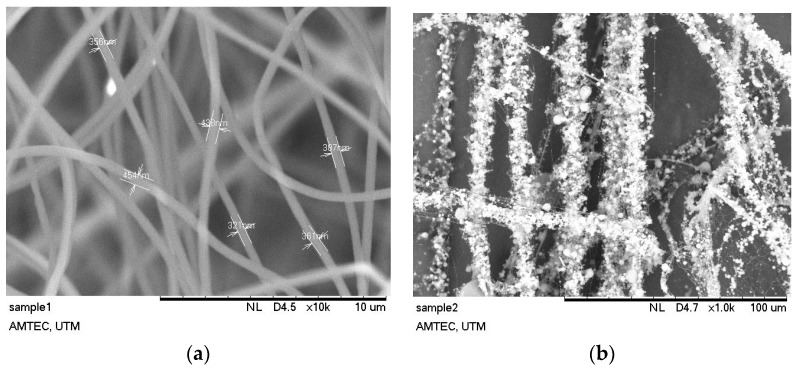
Fiber diameter for: (**a**) SP/e-spunCL15; and (**b**) SP/e-spunCL10.

It is observed that the overall membrane characteristics was increased from SP/e-spunCL10 to SP/e-spunCL15 and the selectivity was dramatically decreased from the SP/e-spunCL10 to SP/e-spunCL25 membranes. The selectivity was increased after adding electrospun fibers with 0.15 wt % of Cloisite loading and decreased when Cloisite content was further increased to 0.25 wt %. The reduction of the value was due to the decrease in proton conductivity that is more significant compared to the methanol permeability. As a result, the maximum overall membrane characteristics was recorded at 0.15 wt % of Cloisite (SP/e-spunCL15), which seems to be the best membranes compared to the other SP/e-spunCL samples. Methanol permeability decreased as the diameter of fibers decreased and became more packed, as performed by SP/e-spunCL15. The fiber diameter of SP/e-spunCL15 is shown in Figure 7a, while the beaded electrospun fibers of SP/e-spunCL10 is shown in Figure 7b, and the summary for average fiber diameters of all samples is tabulated in Table 3.

Fibers with diameters of 386.17–67,680.00 nm were obtained from the electrospinning processed with 20 cm of distance from needle tip to screen collector and constant voltage applied of 22.5 kV. The concentration varied as 0.05, 0.10, 0.17, 0.25 and 0.30 wt %. The variation of fibers structure due to different concentrations can also be explained by the relation between viscosity and surface tension. The viscosity increased when the concentration increased. As the surface tension caused by high voltage supplied tried to reduce the surface area unit per mass, the formation of beads occurred [17]. Viscoelastic forces prevented the formation of beads and allowed the smooth fibers to form.

The lesser is the continuity of fiber, the higher methanol permeation through the membrane, as shown in SP/e-spunCL10. The fibrous structure with good dispersion of Cloisite and exfoliated the membrane surface which not only acted as constraint for methanol to pass through but also created smaller and windier paths in preventing methanol crossover problem. This statement is proven by the value of selectivity, of which SP/e-spunCL15 had the highest of 99.34 × 10^4^. 

**Figure 8:**
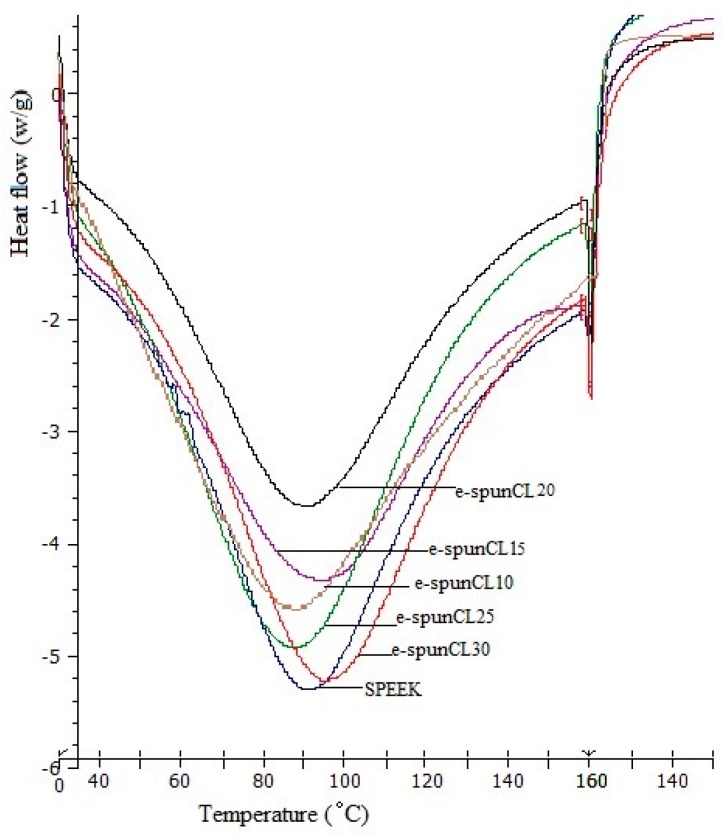
DSC curves heating for SP/e-spunCL and SPEEK membranes.

Further investigation were carried out to reiterate the characteristics of water uptake thermally. Since the TGA results showed the degradation temperature of SPEEK is above 200 °C, the DSC experiments were all handled in the temperature range 50 to 275 °C, which corresponds to the plotted curves in Figure 8. All samples showed the endothermic peak during heating range 85–98 °C due to absorbed moisture.

*T*_g_ can be observed as a peak that is often misread as melting peak or the substantial endorthermic peak of *T*_g_ can also appear in base line [32]. The endothermic peak that appeared (near 160 °C) after the absorbtion of water was caused by the glass transition of the amorphous SPEEK.

**Figure 9:**
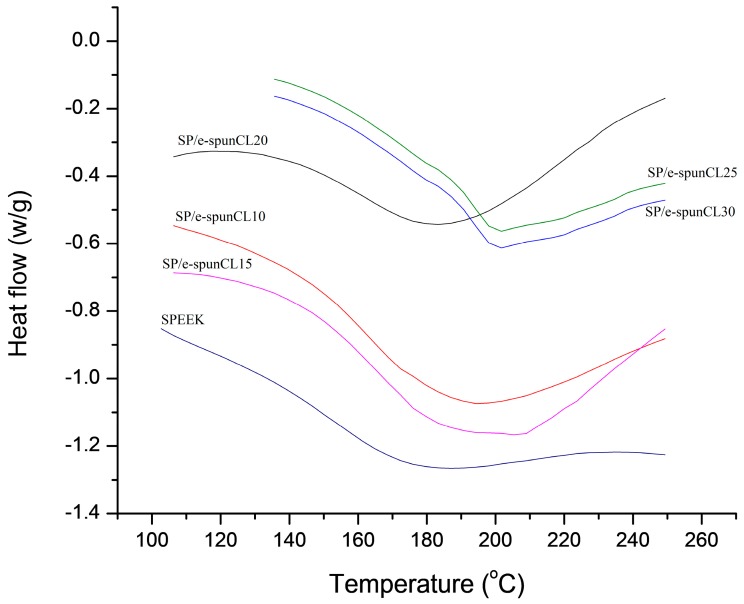
DSC curves reheating for SP/e-spunCL and SPEEK membranes.

It can be explained that *T*_g_ of SPEEK and the SP/e-spunCL samples lies in the range of 150–164 °C. From the comparison of Figure 8 and Figure 9, it can be interpreted that the associated moisture is removed during reheating at the range of temperature around 85–98 °C. Figure 9 shows the *T*_g_ values of all samplesobtained after reheating process, while Table 4 shows the *T*_g_ according to reheat curves. 

The *T*_g_ value of SPEEK (150.20 °C) increases with the incorporation from different concentration of SP/e-spunCL (150.20 to 164.00 °C). The introduction of cloisite fibers into the SPEEK polymer matrix increases *T*_g_ by as much as 13.8 °C for e-spunCL30, 10.13 °C for SP/e-spunCL25, 6.47 °C for SP/e-spunCL15, 2.8 °C for SP/e-spunCL20 and 0.8 °C for e-spunCL10.

From the results, it should be noted that, when the SP/e-spunCL fibers content increases, it gives only small effect to thermal stability (*T*_d_ and *T*_g_), whereas it improves tensile strength significantly. The tensile strength and Young’s modulus of all membrane samples are shown in Table 5. It can be discussed that the notable decrease in tensile strength of SP/e-spunCL25 and SP/e-spunCL30 membranes might be due to the significant exfoliation of SP/e-spunCL fibers or it can be said that the concentration of the SP/e-spunCL higher than that of SP/e-spunCL15 can cause the weak interaction of organic (SPEEK)–inorganic (Cloisite) thus made the contents of Cloisite no longer work as helpful filler but as a defect factor instead. To support the statement, the result from the previous study on composite membranes of Nafion/MMT by Jung reported a similar trend [33].

Many definitions have been made to describe the correlation between hydrophilic polymer and water. In general, the states of water are known as bound water, non-freezing water and free water [34]. Bound water can be recognized when there are small amounts of water entrapped and connected with polar and ionic group that exist in polymer chain. It occurs when absorption of water increased at certain volume, and then, the polar and ionic groups become saturated. The level of bound water relying on the content of ionic groups and the polarity exist in polymer itself [35].

Free water is known as water that has similar characteristic with bulk water and also has the same phase transition, that is 0 °C [35]. Free water can also be found in free space in membrane and crystallizes at higher temperature comparing to bound water. The free water also happens because of the weak interaction of chains within the polymer. Non-freezing bound water is water that has undefined phase transition and different from free water, non-freezing bound water happens because of the strong chains interaction in polymer [36].

Table 6 summarizes the states of water according to heat of melting value (Hfree). The differential scanning calorimetric (DSC) curves correspond to determine the type of water content of SP/e-spunCL10, SP/e-spunCL15, SP/e spunCL20, SP/e-spunCL25 and SP/e-spunCL30 and SPEEK membranes. The test is crucial to study the ability of the membranes in upholding water to provide a high proton conduction.

Since the endothermic peaks in DSC ice-melting diagrams are attributed to the freezable water, for example, freezable bound water and free water, the amount of freezable water in the nanocomposite membranes can be estimated from DSC profile [37]. The endothermic peaks correspond to the freezing free and freezing bound water. The melting enthalpy is obtained through integration and normalization in unit of J·g^−1^ of the swollen membrane. The latent heat of water, 333 J·g^−1^, was taken for the calculation. Based on the calculation, the free water and non-freezing bound water in water uptake are summarized in Table 6.

Only one peak at temperature around 0 °C is observed for all the samples. Hence, the peak is considered to happen from free water. Bound water is suggested to happen due to the hydrogen bond of –SO_3_H in SPEEK polymer chain. 

The methanol permeability and proton conductivity of SP/e-spunCL membranes notably e-spunCL15 were improved as compared to Nafion112^®^ commercial membrane. It can be discussed from involving several factors that give rise to a better SP/e-spunCL membrane characteristics: (1) good dispersion of Cloisite with exfoliated surface membrane; and (2) sufficient water uptake. Table 6 shows the composition of water for all membrane samples.

It is natural for a membrane with low water uptake to perform low methanol permeability. It is generally agreed that methanol permeability increases with the amount of soaked water, but it dominantly occurs via free water (freezing water) inside the interconnected membrane structure channels and insignificantly via non-freezing bound water associated with the ionic sites. As shown in Table 6, SP/e-spunCL15, SP/e-spunCL25 and SP/e-spunCL30 nanocomposite membranes exhibited low amount of free water content and hence low methanol permeability is not surprising. The clearly different values of the three SP/e-spunCL membranes with Nafion^®^ has resulted a significant drop of methanol permeability value from Nafion^®^. The low methanol permeability in SP/e-spunCL membranes can be related to the statement that a membrane with low water uptake has low methanol permeability. Methanol only permeates through free water inside the membrane structure channel (central space) [38].

On the other hand, an increase in the proton conductivity of SP/e-spunCL15 nanocomposite membrane can be interpreted in the following way. The absorbed water molecules are present mostly in the ionic cluster domains and ionic cluster channels. In particular, in the ionic cluster channels, the water molecules exist in two different forms. One is the protonated water (mostly non-freezing bound water) that is bound strongly to the ionic site. The other is free water that occupies the central space free from the influence of the ionic sites. The proton transfer through the ionic cluster channel occurs by two different mechanisms: (1) near the channel wall via the bound water, in which proton is transported by the Grotthuss mechanism, hopping from one ionic site to the other; and (2) via free water by vehicle mechanism, in which proton is facilitated by the water molecules moving through the interconnected central channel space. However, the contribution from the Grotthuss mechanism is more essential. Results presented in Table 6 for SP/e-spunCL15 show the highest value for non-freezing water. Thus, water retention capacity of the nanocomposite membrane was enhanced due to the presence of electrospun fibers with good dispersion of Cloisite, which was expected to reduce the dehydration of membrane. Therefore, it can be said that the enhanced proton conductivity of the SP/e-spunCL15 nanocomposite membrane is attributed to the water retention capability. From these results, it is clear that the incorporation of appropriate amounts of electrospun SPEEK/Cloisite15A^®^ fibers has synergistic effect to lower the methanol permeability and increasing proton conductivity. 

**Figure 10:**
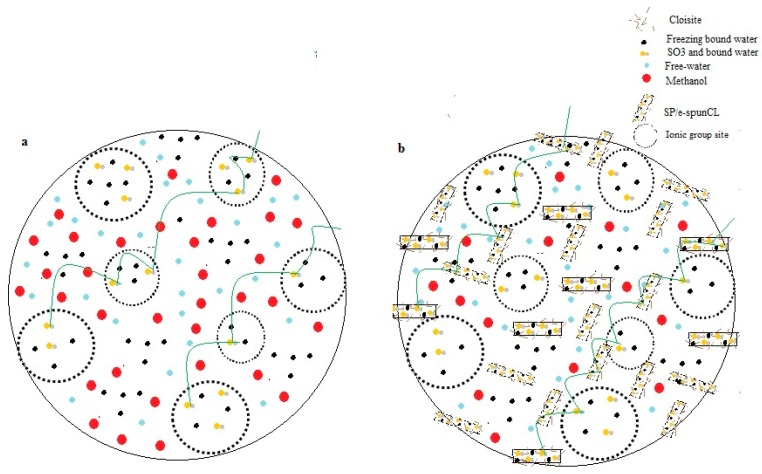
Transport Model of proton and methanol in: (**a**) parent SPEEK; and (**b**) SP/e-spunCL membranes.

This condition has given a significant benefit to SP/e-spunCL membranes. Figure 10a,b shows the model of methanol and proton mobility in parent SPEEK and SP/e-spunCL membranes, respectively.

Figure 10a shows the typical SPEEK membrane without addition of electrospun fibers. It can be seen that, without having any constraint, methanol can easily pass through the channel in SPEEK membrane together with the high amount of free water. Hence, it will result in a membrane with high methanol permeability and possibly cannot curb methanol crossover problem. As for the membrane in Figure 10b, there is the impact of the addition of electrospun fibers which make the methanol pathway become smaller and winding. This phenomenon can also explain the result shown in Table 6, in which electrospun fibers had lower methanol permeability compared to SPEEK membrane and Nafion^®^.

The new configuration of PEM with addition of electrospun fibers is also responsible for the increase in proton conductivity. The Grotthus mechanism (proton hopping) played an important role in bringing the proton from one site to another [39]. The proton was delivered through ionic cluster channel (free water and SO_3_ that linked with non- freezing bound water). It is desirable for a membrane to have high value of bound water (so called non-freezing bound water) since it is a crucial element in delivering protons. To prove the statement, it can be observed that SP/e-spunCL15 showed the highest proton conductivity and also the highest non-freezing bound water. The existence of Cloisite is another factor that help to reduce the hydration in membrane by retaining water [40]. Water retention capability of Cloisite has successfully enhanced the proton conductivity of the SP/e-spunCL membranes.

## 4. Conclusions

A novel SP/e-spunCL membrane is synthesized and characterized. Water uptake, thermal stability and thermal degradation temperature are increased significantly due to the incorporation of electrospun fibers into SPEEK polymer matrix. The impact of temperature on proton conduction can be related to the stimulation process of proton transport, water diffusion and directional change of polymer chains [37]. The SP/e-spunCL15 exhibited the highest proton conductivity of 12.12 ± 0.2837 mS·cm^−1^ with highest non-freezing bound water of 10.60%. The prepared SP/e-spunCL15 membrane had good thermal stability and exhibited promising features as an optimum PEM for DMFC.

## Figures and Tables

**Table 1:** The designation of membrane samples.

Samples	Voltage (kV)	Distance (cm)	Designation
0.10 wt % electrospun SPEEK/Cloisite membrane	22.5	20	SP/e-spunCL10
0.15 wt % electrospun SPEEK/Cloisite membrane	22.5	20	SP/e-spunCL15
0.20 wt % electrospun SPEEK/Cloisite membrane	22.5	20	SP/e-spunCL20
0.25 wt % electrospun SPEEK/Cloisite membrane	22.5	20	SP/e-spunCL25
0.30 wt % electrospun SPEEK/Cloisite membrane	22.5	20	SP/e-spunCL30

**Table 2:** Thermal degradation and char yield of the SP/e-spunCL and SPEEK membranes.

Membrane	First Weight Loss (%)	Second Weight Loss (%)	Third Weight Loss (%)	*T*d_1_ (°C)	*T*d_2_ (°C)	*T*d_3_ (°C)
SP/e-spunCL10	17.85 ± 0.76	12.70 ± 0.98	15.77 ± 0.76	192.1 ± 0.77	389.1 ± 0.70	602.1 ± 0.77
SP/e-spunCL15	16.08 ± 0.56	15.26 ± 0.87	15.74 ± 0.85	201.1 ± 0.87	398.1 ± 0.80	610.1 ± 0.86
SP/e-spunCL20	17.44 ± 0.45	13.69 ± 0.08	15.72 ± 0.07	196.1 ± 0.06	395.1 ± 0.08	606.1 ± 0.06
SP/e-spunCL25	12.61 ± 0.67	14.21 ± 0.65	16.62 ± 0.64	208.1 ± 0.56	406.1 ± 0.56	613.1 ± 0.58
SP/e-spunCL30	9.44 ± 0.34	15.31 ± 0.34	17.95 ± 0.32	209.1 ± 0.34	410.1 ± 0.38	617.1 ± 0.38
SPEEK	20.56 ± 0.23	14.41 ± 0.12	11.12 ± 0.34	163.1 ± 0.45	388.1 ± 0.89	564.1 ± 0.67

**Table 3:** Fibers diameters of electrospun SPEEK/Cloisite fibers.

Concentration (wt %)	Fiber Diameter, nm
SP/e-spunCL10	67,680.0
SP/e-spunCL15	429.2
SP/e-spunCL20	386.17
SP/e-spunCL25	495.4
SP/e-spunCL30	9257.0

**Table 4:** Glass transition temperatures of SP/e-spunCL samples and SPEEK.

Samples	*T*_g_ (°C)
SP/e-spunCL10	151.00
SP/e-spunCL15	156.67
SP/e-spunCL20	153.00
SP/e-spunCL25	160.33
SP/e-spunCL30	164.00
SPEEK	150.20

**Table 5:** The tensile properties of SP/e-spunCL membranes.

Sample	Tensile Strength (MPa)	Young’s Modulus (MPa)
SP/e-spunCL10	29.97 ± 0.78	2743.79 ± 0.56
SP/e-spunCL15	36.35 ± 0.97	3640.74 ± 0.98
SP/e-spunCL20	33.40 ± 0.74	1713.62 ± 0.35
SP/e-spunCL25	28.58 ± 0.67	2681.99 ± 0.37
SP/e-spunCL30	28.61 ± 0.86	1626.70 ± 0.27

**Table 6:** Composition of water type in SP/e-spunCL10, SP/e-spunCL15, SP/e-spunCL20, SP/e-spunCL25, SP/e-spunCL30.

Sample	Total Water (%)	ΔH_f_ Normalized (J·g^−1^ Sample) ^a^	ΔH_f_ Per Mass Water (J·g^−1^ Water) ^b^	Freezing Water/Total Water (%) ^c^	Non-freezing Water/Total Water (%)	Freezing Water/Sample (%)	Non-freezing Water/Sample (%)
SP/e-spunCL10	24.56	67.04	272.96	81.97	18.03	20.13	4.43
SP/e-spunCL15	25.87	50.84	196.52	59.01	40.99	15.27	10.60
SP/e-spunCL20	30.00	70.15	233.83	70.22	29.78	21.07	8.93
SP/e-spunCL25	20.00	57.55	287.75	86.41	13.59	17.28	2.72
SP/e-spunCL30	19.00	59.76	314.52	94.45	5.55	17.95	1.05
SPEEK	26.76	85.46	319.35	95.90	4.10	25.66	1.10
Nafion^®^112	21.43	64.19	299.53	89.95	10.05	19.28	2.15

^a^ obtain from the DSC measurement. ^b^ ΔHf per mass water (J·g^−1^ water) = ΔHf normalized (J·g^−1^ sample)/total water × 100 (%). ^c^ Freezing water/total water (%) = ΔHf per mass water (J·g^−1^ water)/melting enthalpy of pure water, i.e., 333 J·g^−1^.
